# Mill dams impact microbiome structure and depth distribution in riparian sediments

**DOI:** 10.3389/fmicb.2023.1161043

**Published:** 2023-06-29

**Authors:** Jinjun Kan, Erin K. Peck, Laura Zgleszewski, Marc Peipoch, Shreeram Inamdar

**Affiliations:** ^1^Stroud Water Research Center, Avondale, PA, United States; ^2^University of Delaware, Plant and Soil Sciences, Newark, DE, United States

**Keywords:** fragmented rivers, legacy sediments, prokaryotes and fungi, vertical distribution, dam removal

## Abstract

**Introduction:**

Damming has substantially fragmented and altered riverine ecosystems worldwide. Dams slow down streamflows, raise stream and groundwater levels, create anoxic or hypoxic hyporheic and riparian environments and result in deposition of fine sediments above dams. These sediments represent a good opportunity to study human legacies altering soil environments, for which we lack knowledge on microbial structure, depth distribution, and ecological function.

**Methods:**

Here, we compared high throughput sequencing of bacterial/ archaeal and fungal community structure (diversity and composition) and functional genes (i.e., nitrification and denitrification) at different depths (ranging from 0 to 4 m) in riparian sediments above breached and existing milldams in the Mid-Atlantic United States.

**Results:**

We found significant location- and depth-dependent changes in microbial community structure. Proteobacteria, Bacteroidetes, Firmicutes, Actinobacteria, Chloroflexi, Acidobacteria, Planctomycetes, Thaumarchaeota, and Verrucomicrobia were the major prokaryotic components while Ascomycota, Basidiomycota, Chytridiomycota, Mortierellomycota, Mucoromycota, and Rozellomycota dominated fungal sequences retrieved from sediment samples. Ammonia oxidizing genes (*amo*A for AOA) were higher at the sediment surface but decreased sharply with depth. Besides top layers, denitrifying genes (*nos*Z) were also present at depth, indicating a higher denitrification potential in the deeper layers. However, these results contrasted with *in situ* denitrification enzyme assay (DEA) measurements, suggesting the presence of dormant microbes and/or other nitrogen processes in deep sediments that compete with denitrification. In addition to enhanced depth stratification, our results also highlighted that dam removal increased species richness, microbial diversity, and nitrification.

**Discussion:**

Lateral and vertical spatial distributions of soil microbiomes (both prokaryotes and fungi) suggest that not only sediment stratification but also concurrent watershed conditions are important in explaining the depth profiles of microbial communities and functional genes in dammed rivers. The results also provide valuable information and guidance to stakeholders and restoration projects.

## Introduction

Human civilizations originated along big rivers and their deltas, and human activities have had a significant impact on the integrity and function of rivers and their watersheds. Damming is one such activity that has substantially fragmented and altered riverine ecosystems worldwide (Zarfl et al., 2015; Anderson et al., 2018; Belletti et al., 2020; Maavara et al., 2020). In the United States (US), starting in the 1700s, thousands of dams were built by early European settlers for harnessing water power for mills, and were widely distributed on streams and rivers across the eastern United States (Walter and Merritts, 2008; Merritts et al., 2011). Although a majority of the dams have breached or are under consideration for removal due to safety or habitat considerations (Tonitto and Riha, 2016; Foley et al., 2017; Magilligan et al., 2017), more than 14,000 dams still exist across the Northeast United States (Martin and Apse, 2011). While the impacts of dams on fluvial geomorphology and sediment transport (Csiki and Rhoads, 2010; Rodriguez et al., 2020), hydrologic connectivity (Poff et al., 2007; Magilligan et al., 2016), biogeochemical processing (Maavara et al., 2020; Zhang et al., 2021; Inamdar et al., 2022), and aquatic habitat (Barbarossa et al., 2020; Pal et al., 2020) are increasingly recognized, much less is known about how dams and fragmented river systems affect the structure and functions of microbial communities.

Dams alter both the in-channel and the riparian environment in riverine systems. Coupled with accelerated sediment erosion from widespread land clearance and agriculture across the United States (Costa, 1975; Meade, 1982), milldams resulted in large deposits of fine-grained (silt and clay) legacy sediments that formed tall riparian terraces upstream of the dams and with lower floodplains downstream (Walter and Merritts, 2008; James, 2013). Upstream riparian terraces can be many meters tall and are typically the height of the milldams (Merritts et al., 2011). Milldams also alter the hydrologic environment with high stream and groundwater levels upstream (typically equal to the height of the dam) and lower levels and drier conditions downstream (Sherman et al., 2022). The high-water levels and wet and stagnant conditions upstream of the dams promote hypoxic and anoxic conditions in stream and riparian sediments with consequences for carbon and nitrogen biogeochemistry and cycling (Inamdar et al., 2020, 2022; Hripto et al., 2022; Peck et al., 2022, 2023). In contrast, when dams are removed, upstream stream and groundwater levels drop rapidly resulting in drained and oxic riparian sediments (Lewis et al., 2021) that are susceptible to fluvial and subaerial erosive processes (Wolman, 1959; Fox et al., 2016; Gellis et al., 2017). Recent studies have shown that the fine sediment particles and associated nutrients (both dissolved and particulate forms) can serve as important sources to annual sediment and nutrient export to downstream aquatic ecosystems such as the Chesapeake Bay (Gellis et al., 2017; Cashman et al., 2018; Miller et al., 2019; Jiang et al., 2020; Lutgen et al., 2020).

Microorganisms are indicators for river and terrestrial ecosystem functions because they are the major drivers for the most biogeochemical cycles of nutrients globally. In addition to hydrological and morphological discontinuities, river fragmentation and damming is likely to disturb these nutrient-cycling pathways by reassembling and reconstructing the microbial compositions. In a previous molecular survey, our results showed that heterotrophic bacteria in the milldam-associated legacy sediments were different from other soil environments (Sienkiewicz et al., 2020). Distinct depth profiles of these microbes were also observed in their detailed structure composition and microbial activities: higher carbon respiration in the surface sediment while lower microbial enzyme activities in deeper layers (Weitzman et al., 2014; Weitzman and Kaye, 2017). Our studies further confirmed this observation by showing very low amended or unamended denitrification enzyme activity (DEA; Peck et al., 2022), although higher denitrification genes were found in deeper layers (Sienkiewicz et al., 2020). By quantifying in-stream denitrification rates above and below milldams, Hripto et al. (2022) concluded that milldams that were filled to capacity with sediments had limited effects on nitrogen removal and transport in stream ecosystems. However, there is still some debate on whether legacy sediments serve as sources or sinks of nutrients (N, P, etc.); and addressing this question is crucial for the decision-making process on sediment removal and stream restoration (Inamdar et al., 2020).

These disagreements and arguments highlight critical knowledge gaps and a lack of understanding of how river fragmentation with dams impacts ecosystem functions, which are primarily manipulated by microorganisms (e.g., Graham et al., 2016). Due to the backup of stream water above dams, we hypothesize the elevated groundwater and anoxia enhance the depth stratification of microbial communities in the sediments. This is particularly so for milldams which have been in place since the mid-1700s—representing more than 200 years of continuous saturation and anoxia in riparian sediments. When the dams are removed, riparian sediments drain rapidly and become oxic—representing an abrupt or instantaneous (at the geologic and ecological time scales) change in the biogeochemical and microbial environment. This sudden change likely alters the nitrifying and denitrifying processes and associated microbial communities (e.g., Lewis et al., 2021). It is important to understand how these hydrologic and biogeochemical changes pre and post dam removals affect the microbial community structure and functions and their evolution over time.

In this study, we collected sediment cores from riparian sediment terraces above three milldams in the mid-Atlantic United States: two with standing milldams (Roller and Cooch) and one recently-breached dam site (Krady). Depth profiles (0–4 m) of community structures of bacteria/archaea and fungi were characterized with high throughput sequencing of 16S rRNA genes and ITS regions. Nitrification and denitrification genes were used to quantify the functional measure in the sediment cores. We aim to further our understanding of how dam-fragmented riverine systems impact microbial assembly and function.

## Materials and methods

### Experimental design and sample collection

**Figure 1:**
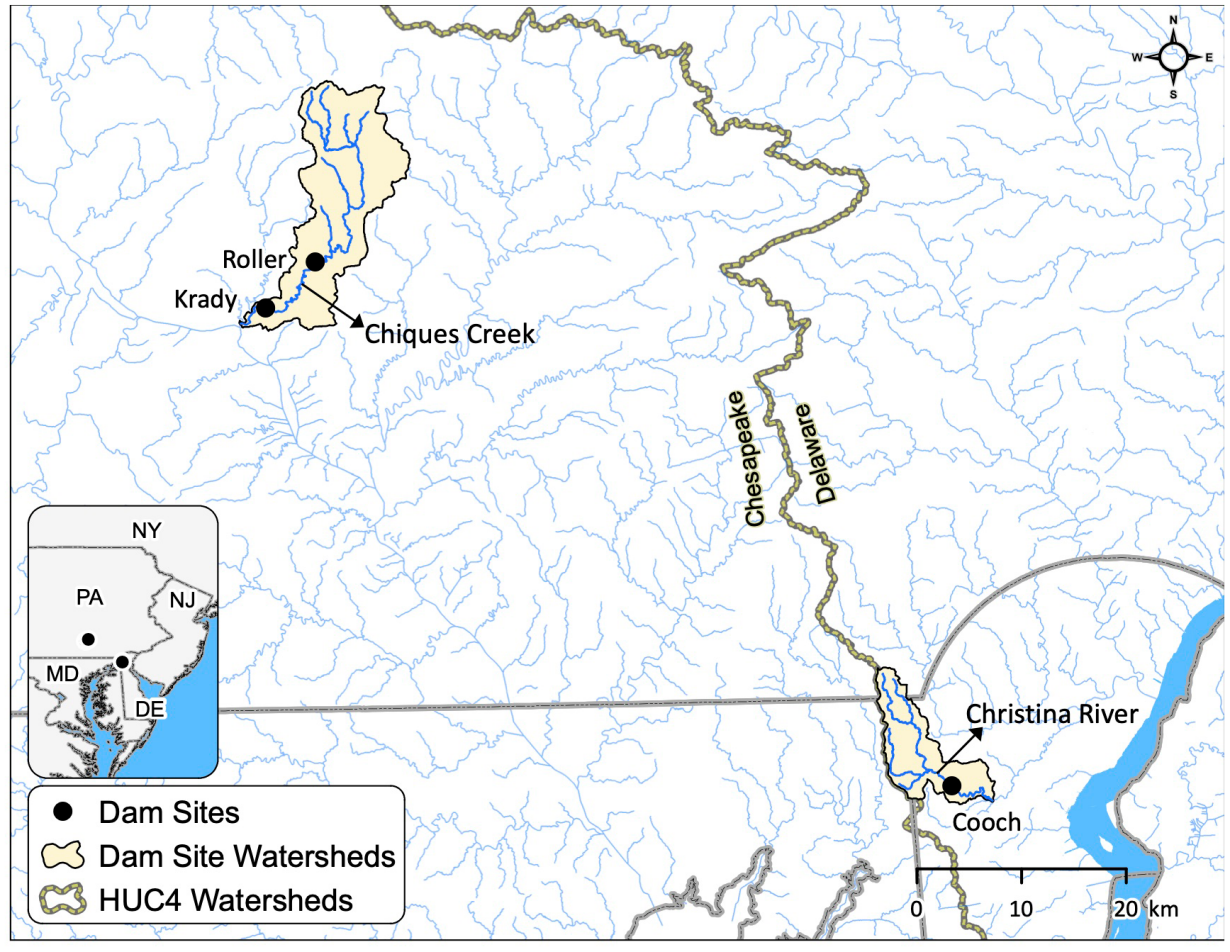
Maps showing the sampling locations for sediment collection. Krady and Roller dam sites are located at Chiques Creek in Pennsylvania (Chesapeake watershed), and Cooch milldam site is located at Christina River in Delaware (Delaware watershed).

Three low-head milldam sites—Krady, Roller and Cooch were selected for this study, Krady dam was breached and removed in July 2018, while Roller and Cooch are still standing and largely intact. Krady (1.5 m tall) and Roller (2.4 m tall) milldams are both on Chiques Creek (Krady is 10 km downstream of Roller) in Pennsylvania draining into the Chesapeake Bay, while Cooch milldam (~4 m tall) is located on Christina River in Delaware that drains into the Delaware Bay (Figure 1). All of these dams are fairly representative of the types of milldams constructed in the mid-Atlantic United States (Walter and Merritts, 2008). Krady and Roller milldams were constructed in mid-1700s (exact date unknown) whereas the Cooch milldam was built in 1792 (Inamdar et al., 2022). Thus, for Roller and Cooch, the riparian sediments immediately upstream of the dam have been in a saturated and hypoxic/anoxic condition for more than 200 years. For Krady, the dam removal occurred in 2–4 h and riparian sediments drained within days (Lewis et al., 2021). Riparian sediments above all the dams are primarily fine-grained with lenses of coarse grained (sand) sediments (Peck et al., 2022, 2023). Carbon dating of buried organic material at depths of 3–4 m suggest that much of the riparian legacy sediments were deposited in the past 200–300 years (Peck et al., 2022).

Chiques creek drains a primarily agricultural watershed while the Christina River has a mixed landuse (urban and agriculture) above the Cooch milldam (Inamdar et al., 2022; Peck et al., 2022). Above the Krady dam site on the Chiques Creek, the major land uses are 68% agricultural, 13% forested, 11% residential, and 7% grassland (USGS National Landcover Database, 2011; Lewis et al., 2021). The Cooch dam site is a more urbanized site near Newark in Delaware, consisting of 47% urban/developed, 30% forested, and 23% agricultural land (USGS National Landcover Database, 2011; Peck et al., 2022). Riparian sediment and groundwater biogeochemistry above Cooch milldam on Christina River is also affected by road salt (NaCl) inputs from a large interstate highway (Inamdar et al., 2022). The Chiques watershed is mostly dolomite/limestone (rich in Calcium and magnesium) and shale, while the Christina River basin is dominated by gabbro and gneiss. Chiques Creek and Christina River have a temperate weather, with similar mean annual air temperatures (15.5 and 12.2°C, respectively) and precipitation amounts (104 and 114 cm, respectively; NOAA 2021). Both riparian areas are forested with dominant tree species including sugar maple (*Acer saccharum*), black walnut (*Juglans nigra*), and American sycamore (*Platanus occidentalis*).

Riparian sediments upstream of all milldams are about as thick as the heights of the dams (Lewis et al., 2021; Inamdar et al., 2022) and were sampled for this study. A total of five riparian sediment cores were collected using a hand-operated auger to a depth of refusal (~3–4 m): two from Krady dam site (KMW1B and KMW3B), one from Roller milldam (RMT1W1), and two from Cooch milldam (CMT1W1 and CMT2W1). All of these cores were collected within 10 m from the stream edge and detailed sampling sites, procedures, and chemical analyses can be found in previous publications (Inamdar et al., 2022; Peck et al., 2022, 2023; Sherman et al., 2022). The cores were segmented to different depths on site, stored in air-tight Whirl-Pak® bags and transported to the lab on ice (4°C; Table 1). Samples from each depth were sent to labs for chemical analysis and measurements (Supplementary Table S1), and a subset of samples were saved in freezer (−80°C) for molecular analyses (see below).

**Table 1:** Location of sampling sites, sampling dates, depth, species richness, diversity and functional genes for the depth sediment profiles.

Sites	River/Sampling date	Dam and location	Sample ID	Depth (mbs)	Bacteria/archaea (16S)			Fungi (ITS)			AOA (10^4 copies/g) ± SD	nosZ (10^4 copies/g) ± SD
					Chao1	Shannon	Faith PD	Chao1	Shannon	Faith PD		
KMW1B	Chiques Creek/Nov 6, 2019	Krady dam/40°04'08.2"N, 76°29'58.7"W	KMW1B_1	0.30	2040.0	10.06	96.09	1086.6	7.68	130.71	755.10 ± 37.82	116.80 ± 20.60
			KMW1B_3	0.91	2082.2	10.01	104.30	674.4	7.77	95.17	114.53 ± 18.23	5.30 ± 0.83
			KMW1B_6	1.68	2158.8	10.07	117.56	721.3	7.07	94.16	75.27 ± 11.76	12.95 ± 1.42
			KMW1B_7	1.98	2264.0	10.04	139.49	178.0	6.33	43.33	16.13 ± 1.58	14.17 ± 1.02
			KMW1B_8	2.44	2218.5	9.97	129.38	372.1	6.41	57.51	2.34 ± 0.25	1.15 ± 0.16
			KMW1B_10	2.90	2185.5	9.80	126.42	308.9	7.20	51.46	0.89 ± 0.03	0.39 ± 0.080
KMW3B	Chiques Creek/Nov 6, 2019		KMW3B_0	0	2151.2	10.16	100.20	1434.5	8.28	165.86	ND	119.93 ± 11.10
			KMW3B_1	0.30	2008.0	10.03	97.58	1192.4	8.37	145.39	ND	110.23 ± 13.60
			KMW3B_2	0.61	1942.2	9.90	100.46	697.1	7.93	105.61	7.38 ± 0.43	99.59 ± 8.57
			KMW3B_4	1.22	1978.2	9.87	107.42	659.4	6.65	95.86	25.15 ± 2.66	25.97 ± 2.28
			KMW3B_5	1.52	1940.2	9.62	112.46	625.3	7.46	86.81	58.31 ± 3.52	34.33 ± 2.73
			KMW3B_6	1.83	1723.0	9.07	105.01	461.5	7.96	69.47	68.36 ± 18.56	78.24 ± 13.42
			KMW3B_7	2.13	2042.5	9.75	120.28	461.8	7.85	70.91	19.50 ± 3.17	7.94 ± 0.75
			KMW3B_9	2.74	2089.2	9.70	128.21	195.7	5.55	43.59	0.86 ± 0.13	0.44 ± 0.11
												
RMT1W1	Chiques Creek/Aug 30, 2019	Roller dam/40°06'29"N, 76°26'35"W	RMT1W1_0	0	1936.0	10.00	91.56	961.0	6.29	121.85	631.37 ± 224.19	152.83 ± 7.88
			RMT1W1_2	0.61	1126.1	7.00	71.91	271.2	3.01	47.77	40.96 ± 12.23	40.63 ± 14.10
			RMT1W1_4	1.22	1114.2	6.45	78.78	414.5	5.66	69.32	21.23 ± 4.51	101.43 ± 4.33
			RMT1W1_6	1.83	1112.0	6.10	74.76	386.8	7.00	61.65	26.08 ± 6.48	124.98 ± 11.13
			RMT1W1_8	2.44	1158.6	6.33	80.23	486.1	6.18	69.55	29.60 ± 7.72	154.01 ± 15.16
			RMT1W1_10	3.05	819.1	5.32	60.50	345.4	5.44	49.69	1.04 ± 0.68	198.00 ± 12.74
												
CMT1W1	Christina River/Oct 25, 2019	Cooch dam/39°38'44"N, 75°44'33"W	CMT1W1_0	0	1844.3	9.87	87.62	948.4	6.39	132.08	11.67 ± 0.012	195.80 ± 32.08
			CMT1W1_2	0.61	1659.1	8.64	107.77	322.0	4.54	50.81	54.32 ± 1.56	23.15 ± 1.35
			CMT1W1_3	0.91	1610.4	7.60	103.30	245.5	5.52	42.29	6.72 ± 0.11	2.83 ± 0.93
			CMT1W1_5	1.52	1855.1	8.48	104.80	190.1	4.96	32.06	0.26 ± 0.045	1.61 ± 0.34
CMT2W1	Christina River/Oct 25, 2019		CMT2W1_0	0.00	1898.0	9.96	89.92	881.4	6.07	111.77	214.76 ± 38.71	140.32 ± 28.42
			CMT2W1_3	0.91	1739.9	8.12	103.35	230.1	3.17	37.02	2.35 ± 0.65	0.39 ± 0.071
			CMT2W1_6	1.83	1506.2	8.22	93.00	317.2	4.30	46.13	0.22 ± 0.036	11.50 ± 1.60
			CMT2W1_9	2.74	1756.8	8.46	110.07	233.6	2.80	36.23	0.0055 ± 0.0026	0.93 ± 0.21
			CMT2W1_12	3.66	1897.9	8.09	117.24	231.0	3.93	36.16	0.011 ± 0.0092	0.13 ± 0.027

mbs, meter below surface; AOA, ammonia oxidizing archaea; nosZ, nitrous oxide reductase gene; and ND, not detected.

### DNA extraction, high throughput sequencing, and qPCR

Genomic DNA was extracted from sediment samples (0.25 g, wet weight) using DNeasy PowerSoil Pro Kits (Qiagen, Hilden, Germany) following the manufacturer’s instructions. DNA concentrations and purity were measured using an ND-20000 NanoDrop spectrometer (Thermo Fisher Scientific, Waltham, United States).

High throughput sequencing was performed on a total of 29 samples to characterize detailed bacterial/archaeal (16S) and fungal (ITS) communities. Library preparation followed the Sequencing Library Preparation protocols from Illumina.^1^ For bacteria and archaea, the V4 variable region of the 16S rRNA genes was amplified using the forward primer 515f (5′-GTGYCAGCMGCCGCGGTAA-3′; Parada et al., 2016) and reverse primer 806r (5′-GGACTACNVGGGTWTCTAAT-3′; Apprill et al., 2015) following the Earth Microbiome Project protocol (Gilbert et al., 2010). For fungi, the ITS2 region was amplified using the forward primer ITS3-F (5’-GCATCGATGAAGAACGCAGC-3′) and reverse primer ITS4-R (5’-TCCTCCGCTTATTGATATGC-3′; White et al., 1990). PCR contained 25 μL 2x Premix Taq, 1 μL each primer (10 μM), 1 μL bovine serum albumin (BSA), and 50 ng environmental DNA template in a volume of 50 μL. 16S rRNA genes were amplified with following thermocycling program: 5 min at 94°C for initialization; 30 cycles of 30 s denaturation at 94°C, 30 s annealing at 53°C, and 30 s extension at 72°C; followed by 8 min final elongation at 72°C. Fungal ITS regions were amplified with following thermocycling program: 3 min at 95°C for initialization; 33 cycles of 20 s denaturation at 95°C, 20 s annealing at 56°C, and 30 s extension at 72°C; followed by 5 min final elongation at 72°C. Sequencing libraries were prepared by using NEBNext Ultra II DNA Library Prep Kit for Illumina (New England Biolabs, Massachusetts, United States) following manufacturer’s recommendations. High-throughput sequencing was performed at Magigene (Magigene Biotechnology, Guangzhou, China) on an Illumina Nova6000 platform (paired-end 250-bp mode), following the manufacturer’s guidelines. Raw sequencing data obtained in this study are available through the GenBank database under the accession number PRJNA925921.

Nitrogen transformation genes were used to determine functional gene abundance for each depth—ammonia monooxygenase (*amo*A) genes for ammonia oxidizing archaea (AOA) and nitrous oxide reductase (*nos*Z) for denitrifying microorganisms. Previous surveys concluded that AOA predominated the nitrifying microbes in sediment and soil samples (Leininger et al., 2006; Sienkiewicz et al., 2020), therefore, we used AOA to represent the nitrification potential. The primer information, SYBR Green qPCR reactions, and thermal programs were set up following previously described protocols (Kan, 2018; Sienkiewicz et al., 2020). Briefly, ammonia monooxygenase genes (*amo*A) were amplified with Arch-*amo*Af and Arch-*amo*Ar primers (Francis et al., 2005), and nitrous-oxide reductase genes (*no*sZ) were amplified by the primer set: *nos*F (Kloos et al., 2001) and *nos*ZR^1622^ (Throba Ck et al., 2004). Each sample was run in triplicates and 10-fold dilution series were generated from corresponding plasmids (standard curves were shown in Supplementary Figure S2). The copy number per gram of sediment was calculated based on the concentration of plasmid DNA and amplicon size used in the standard curves (Einen et al., 2008; Kan, 2018).

### Data analysis and statistics

Raw Illumina sequences were processed with the QIIME 2 software package (version 2021.11; Bolyen et al., 2019). After demultiplexing, all raw sequence reads were carried out with quality control, denoising, filtering, merging, and chimera removal through q2-DADA2. Amplicon sequence variants (ASVs) were generated, and a Naïve Bayes classifier artifact^2^ was applied to assign the ASVs to taxa at 99% using the Silva classifier 132 (April 10 2018) for 16S rRNA genes, and UNITE version 8.2 (February 20, 2020) for ITS regions.

The ASVs were normalized by rarefaction approach performed with Qiime 2 pipeline, with cutoffs at 94,000 sequences for bacteria/archaea and 53,000 for fungi (coverage >99.7% of the total diversity for both, see Supplementary Figure S1). Normalized and aggregated ASV tables were used to calculate Chao1 richness (Chao, 1984), Shannon diversity index (Shannon, 1948), and phylogenetic diversity (Faith PD; Faith, 1992). Chao1, Shannon, and Faith PD were calculated using the qiime2 q2-diversity plugin: Chao1 and Shannon used the “alpha” method, and Faith PD used the “alpha-phylogenetic” method.^3^

In order to investigate the similarity/dissimilarity and distribution of microbial communities across depths and sites, non-metric multidimensional scaling (NMDS) was conducted using the MDS procedure in SAS/STAT (v9.4, SAS Institute Inc., Cary, NC, United States) based on Bray–Curtis dissimilarity index (Bray and Curtis, 1957). The screen plot suggested that the first two dimensions were sufficient in defining the overall dimensionality of the input data with stress values close to or less than 0.1 (Clarke, 1993). Target or microbial community groups of interest were visually identified based on the screen plots, and similarity analysis between groups (ANOSIM) was performed using the “anosim” functions in the “vegan” package (version 3.6.1; Oksanen et al., 2018) under the R software (version 4.1.2).

Assessing specific taxonomic groups that drove a particular NMDS result was examined by correlating (Spearman rank), the relative abundance of each taxon against the NMDS dimension scores. In a similar manner, the environmental parameters including soil chemistry (Inamdar et al., 2022; Peck et al., 2022) were correlated with NMDS structure by using SAS Version 9.4 (SAS Institute Inc.). Significant correlations (*p* < 0.05) indicate which taxa or environmental variables are driving differences in bacteria/archaea or fungal community structures.

## Results

### Environmental measurements across depth

When compared across all depths, %C, %N, and OM were higher at both surface and deep sediments (Supplementary Table S1). C:N ratios were significantly larger at Cooch than Roller and Krady sites (*p* < 0.01, Supplementary Table S1; Peck et al., 2022). Concentrations of most M3 extracted minerals including P, Ca, Mg, Zn, Cu, B etc. decreased with depth, while concentrations of Mn, Fe, and S were higher in deeper layers. Na concentrations at Cooch dam site were significantly higher than those at Krady and Roller dam site, which were likely attributed to the road salt applications (Inamdar et al., 2022). Nitrate-N concentrations peaked at the surface sediment and varied with depth at all three sites. In contrast, ammonium-N concentrations peaked at deeper sediment depths at all three sites, and these depth trends were significant (*p* < 0.01; Supplementary Table S1; Peck et al., 2022). Unamended DEA rates and amended DEA rates were higher at surface than deep layers across all three sites, while nitrification and mineralization were higher at Krady than Roller and Cooch sites (Supplementary Table S1). Detailed comparison of soil biogeochemistry across depths between these sites were described in Peck et al. (2022).

### Sequence data and diversity

After demultiplexing, a total of 4,195,000 reads were obtained for 16S rRNA genes and a total of 6,602,709 were obtained for ITS regions. Quality control, denoising, filtering, merging, and chimera removal were conducted through q2-DADA2, and resulted in 2,953,026 valid reads for 16S rRNA genes, and 3,766,814 for ITS regions. In order to minimize the sampling effects, the original ASV tables were rarified to a depth of 94,000 sequences per sample for bacteria/archaea, and 53,000 sequences per sample for fungi. Alpha rarefaction analyses were used to document that samples were sequenced to a sufficient depth with coverages over 99.7% of the total diversity (Supplementary Figure S1). After rarefaction, 19,759 unique ASVs for bacteria/archaea and 7,888 unique ASVs for fungi were identified.

From surface to depth, Shannon index for prokaryotes (bacteria and archaea) decreased across all sites, but species richness (Chao1) was higher at top and bottom layers compared to middle layers (except samples from Roller dam; Table 1). Prokaryotic Faith PD increased with depths at both Krady and Cooch dam sites, but decreased at Roller dam. For fungi, both diversity index (Shannon and Faith PD) and species richness showed decreasing trends with depths, where top layers contained the highest unique ASV numbers and fungal diversity (Table 1).

### Microbial compositions across depths at three dam sites

**Figure 2:**
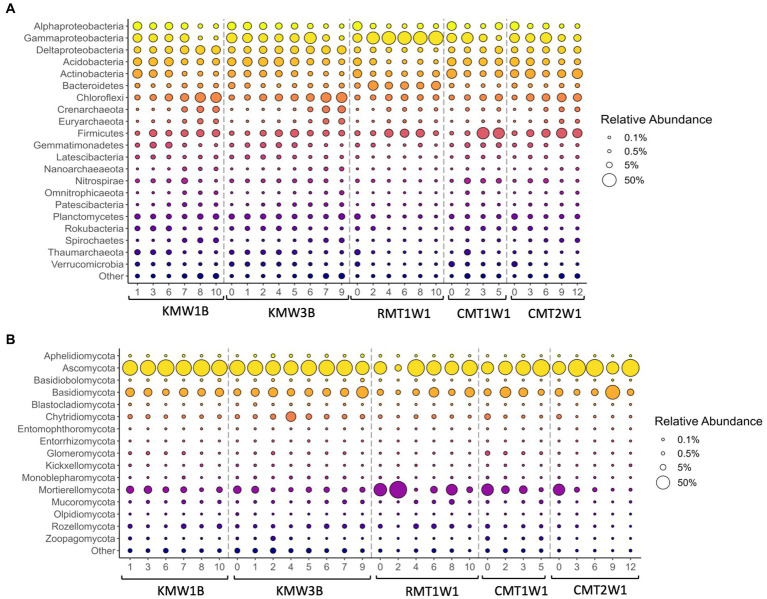
Distribution of major bacterial and archaeal phyla **(A)** and fungi **(B)** across depths. Bubble size represents the relative abundance of each taxa over the total sequence reads. Phyla were included in the “Other” category if they failed to meet two criteria—either that the phylum present at >1% in any sample, or that it was present at >0.1% in all samples. This category also included ASVs that were not identified to the phylum level.

Regarding the community composition, the microbial assemblies across three dam sites are generally similar: more than 19 major bacterial/archaeal phyla were commonly found in the riparian sediments, such as Proteobacteria (mainly subclasses alpha, gamma, and delta), Acidobacteria, Actinobacteria, Bacteroidetes, Chloroflexi, Crenarchaeota, Euryarchaeota, Firmicutes, Gemmatimonadetes, Latescibacteria, Nanoarchaeaeota, Nitrospirae, Omnitrophicaeota, Patescibacteria, Planctomycetes, Rokubacteria, Spirochaetes, Thaumarchaeota, and Verrucomicrobia (Figure 2A). Among them, Actinobacteria and Firmicutes were more abundant at Cooch site while the relative abundances of Deltaproteobacteria, Planctomycetes, and Thaumarchaeota were higher at Krady and Roller sites. Distribution of other major bacterial/archaeal phyla responded to depth across all the sites (Figure 2) and most of them showed a clear trend of increasing or decreasing. Relative abundance of Deltaproteobacteria, Chloroflexi, Firmicutes, Spirochaetes, Crenarchaeota, and Euryarchaeota increased with depth while Alphaproteobacteria, Acidobacteria, Actinobacteria, Gemmatimonadetes, Rokubacteria, Verrucomicrobia, and Thaumarchaeota showed negative correlations with depths (Figure 2). Gammaproteobacteria dominated the total community at Roller mill site (RMT1W1) and reached close to 50% of total community at most layers (Figure 2A).

Based on the relative abundance, Aphelidiomycota, Ascomycota, Basidiomycota, Chytridiomycota, Glomeromycota, Mortierellomycota, Mucoromycota, Rozellomycota, and Zoopagomycota were the major fungal phyla found in the sediments (Figure 2B). For instance, Ascomycota dominated all the samples across sites and depths with relative abundance ranged from 40.23 to 91.49% except the sample from 0.6 m depth at Roller (RMT1W1_2; 7.45%) in which the relative abundance of Mortierellomycota reached 89.74%. Compared to bacteria and archaea, fungal groups did not show clear increasing or decreasing trends with depth, but certain groups of fungi were more abundant in the surface soils, such as Blastocladiomycota, Kickxellomycota, Entorrhizomycota, Olpidiomycota, and Zoopagomycota (Figure 2B). In addition, two phyla, Mucoromycota and Rozellomycota were found more dominant in deep layers (Figure 2B).

Non-metric Multidimensional Scaling plots confirmed spatial patterns across watersheds, sampling sites, and sediment depths (Supplementary Figures S3A,B). Both bacterial and fungal communities across watersheds/sampling sites were separated mainly along NMDS axis 2, while microbial communities within each site were separated primarily by depth along NMDS axis 1 (Supplementary Figure S3). Analysis of Similarity (ANOSIM) showed both bacterial and fungal communities were distinct between streams/watersheds (Chiques Creek—Krady and Roller versus Christina River—Cooch, *p* < 0.05). Further, the compositions of bacteria/archaea and fungi from each dam site (Krady vs. Roller vs. Cooch) were also distinct (Table 2). The two depth profiles from the breached dam site on Chiques Creek—Krady (KMW1B and KMW3B) were different from the existing dam site on the same creek—Roller (RMT1W1).

**Table 2:** ANOSIM results for NMDS separations of microbial communities.

16S bacteria and archaea	KMW1B + KMW3B	RMT1W1	ITS fungi	KMW1B + KMW3B	RMT1W1
					
RMT1W1	*R* = 0.4059		RMT1W1	*R* = 0.3230	
	**p = 0.0015**			**p = 0.0114**	
CMT1W1 + CMT2W1	*R* = 0.5382	*R* = 0.3936	CMT1W1 + CMT2W1	*R* = 0.8900	*R* = 0.8017
	**p = 1e−04**	**p = 0.0056**		**p = 1e−04**	**p = 5e−04**

Significant differences (*p* < 0.05) are shown in bold.

**Figure 3:**
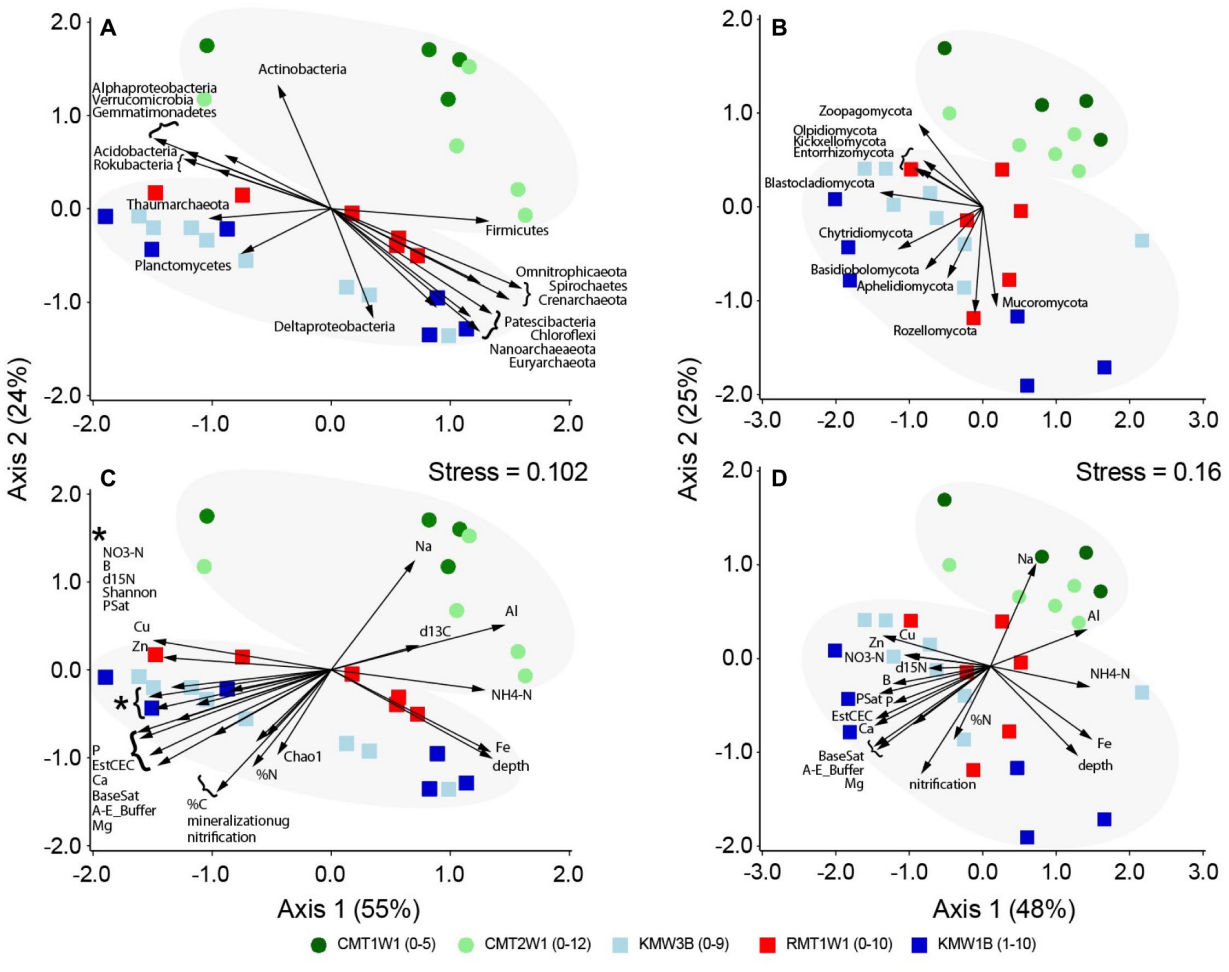
Correlations of microbial groups and environmental parameters with NMDS separation of community structures: **(A)** and **(C)** for bacteria and archaea and **(B)** and **(D)** for fungi. Only significant microbial phyla or environmental parameters (*p* < 0.05) are shown with vectors.

Correlations of microbial taxa with NMDS patterns verified the depth distribution of major bacteria and archaea as shown in Figure 2: Alphaproteobacteria, Acidobacteria, Actinobacteria, Gemmatimonadetes, Rokubacteria, Verrucomicrobia, and Thaumarchaeota dominated in surface layer, and Deltaproteobacteria, Chloroflexi, Firmicutes, Omnitrophicaeota, Patescibacteria, Spirochaetes, Crenarchaeota, and Euryarchaeota were more abundant in deep layers (Figure 3A). Planctomycetes also showed a significant correlation with the microbial distribution but they are more enriched in top layers at Chiques sites (Figure 3A). In contrast, only a few fungal groups showed positive (Mucoromycota and Rozellomycota) or negative correlations (e.g., Blastocladiomycota, Kickxellomycota, Entorrhizomycota, Olpidiomycota, and Zoopagomycota) with soil depths (Figure 3B). Aphelidiomycota, Basidiobolomycota, and Chytridiomycota were found more abundant at Krady and Roller sites compared to Cooch site (Figure 3B).

Except depth and Fe concentration, environmental variables that correlated with microbial distribution (both bacteria/archaea and fungi) primarily differentiated the samples between watersheds, i.e., Chiques vs. Christina (Figures 3C,D). High concentrations of NH_4_-N, Al, and Na occurred at Cooch site, but samples from Krady and Roller sites contained higher NO_3_-N, %C, %N, and minerals (Cu, Zn, B, P, Ca, Mg etc.). Base saturation (BaseSat), saturation percentage (PSat), mineralization, nitrification, species richness, and Shannon diversity index were also higher at Krady and Roller sites than Cooch site (Figures 3C,D).

### Comparison between before vs. after dam removal

**Figure 4:**
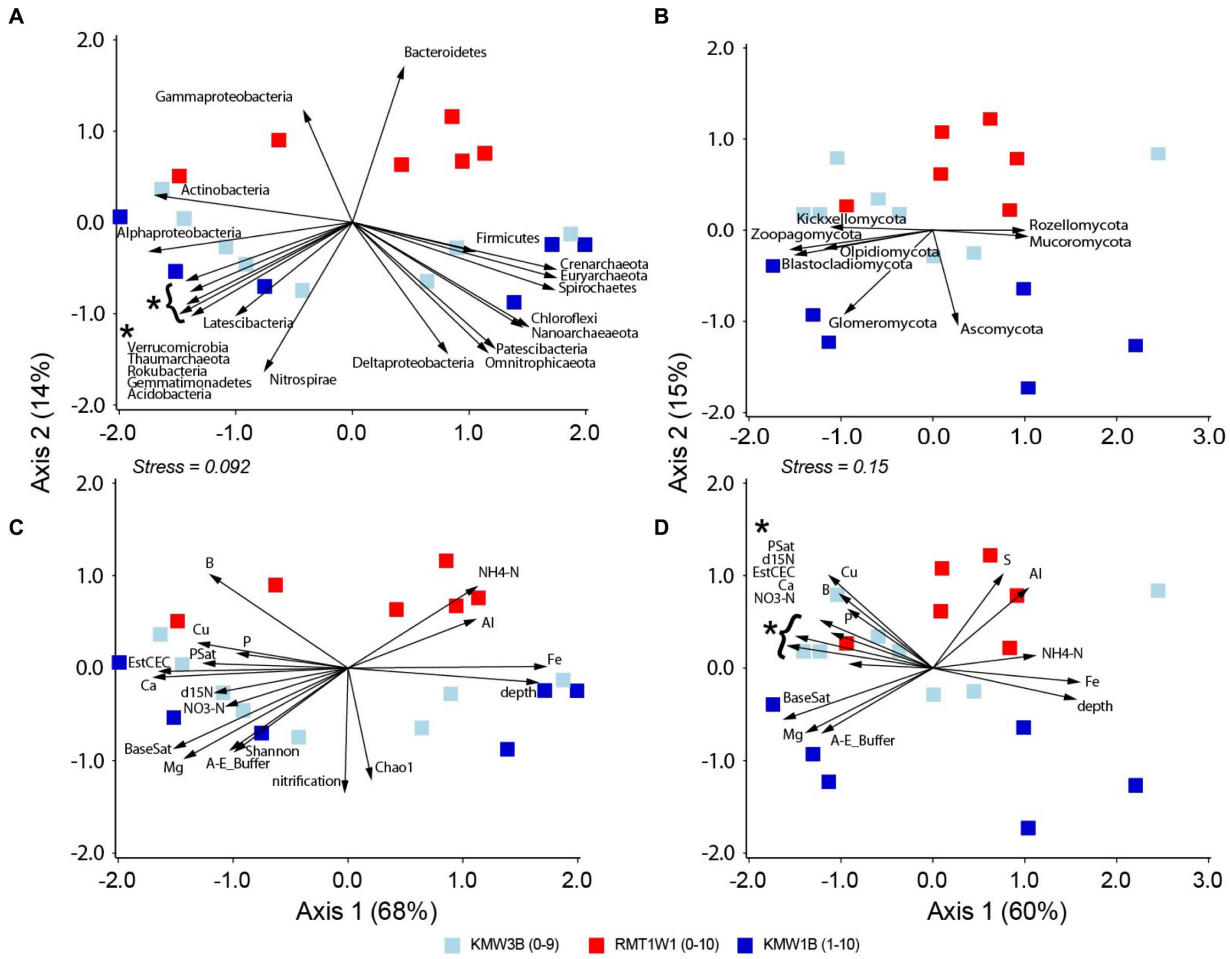
Correlations of microbial groups and environmental parameters with NMDS separation of community structures at Krady and Roller dam sites: **(A)** and **(C)** for bacteria and archaea and **(B)** and **(D)** for fungi. Only significant microbial phyla or environmental parameters (*p* < 0.05) are shown with vectors.

By contrasting samples from Roller (existing dam) and Krady (dam removed) sites (RMT1W1 vs. KMW1B and KMW3B), we were able to compare microbial communities before vs. after dam removal. The results showed both bacteria/archaea and fungi were different between these two sites and a wider separation with depths at Krady than Roller site (Figures 4A,B, Table 2). Samples from Krady site contained high abundances of many bacterial (Alphaproteobacteria, Acidobacteria, Actinobacteria, Nitrospirae, Verrucomicrobia etc.) and fungal phyla (such as Blastocladiomycota, Glomeromycota, Kickxellomycota, Olpidiomycota etc.) in the top layers. For deep layers, abundant bacterial groups including Spirochaetes, Firmicutes and archaea (Crenarchaeota, Euryarchaeota, and Nanoarchaeota) and fungi (Ascomycota, Mucoromycota, and Rozellomycota) were retrieved from Krady sites. In contrast, only Gammaproteobacteria and Bacteroidetes were found more abundant in samples from Roller site (Figure 4A). Environmental measurements also differed between sampling sites: Roller site contained higher NH_4_-N, Al, and S while Krady site had higher NO_3_-N (Figures 4C,D). Samples from Krady sites (after dam removal) also contain higher species richness, microbial diversity as well as nitrification (Figure 4C). Across depths, higher minerals (Ca, Cu, P, etc.) and δ^15^N occurred in surface soils and Fe was more abundant in deeper layers (Figures 4C,D).

### Nitrification and denitrification genes

We quantified AOA and *nos*Z genes to estimate nitrification and denitrification potentials for different layers. In general, nitrifying genes were abundant at the top and decreased with depths except KMW3B site where the AOA peaked at 1.83 m below surface (Table 1). Most denitrifying genes *nos*Z were also found at the top layers but moderate amounts were observed in the middle or bottom layers. At Roller site, *nos*Z genes increased with depths and the highest quantity was at 3 m deep (Table 1). In contrast, the denitrifying genes were low in deeper layers at Cooch site. These results demonstrated that, in addition to depth and oxygen gradients, the vertical distribution of nitrifying and denitrifying microbes was likely influenced by other environmental conditions. For instance, hydrology and soil biogeochemistry at the Cooch site are affected by road salt application from an interstate highway, and the potential impact of which on soil microbial processes is under investigation.

## Discussion

### Spatial distribution across depth and watershed: local land use and concurrent conditions

Given the origin, accumulation process and hydrological interactions, the legacy sediments are distinct geochemically compared to forest wetland or agriculture soils (Walter and Merritts, 2008; James, 2013; Jiang et al., 2020; Lutgen et al., 2020). Typical soil bacteria, archaea and fungi have been found in the sediment samples, such as Acidobacteria, Proteobacteria (Alpha-, Gamma-, and Delta-), Chloroflexi, Nitrospirae, Verrucomicrobia, Crenarchaeota, Euryarchaeota, Basidiomycota, Ascomycota, and among others (Janssen, 2006; Bergmann et al., 2011; reviewed by Fierer, 2017 and the references therein), but the detailed microbiome composition and distribution in dam-associated legacy sediments differed from other soil environments (Koval, 2011; Weitzman et al., 2014; Sienkiewicz et al., 2020). Distinct depth profiles of sediment microbiomes were observed for both prokaryotes and fungi in this study, suggesting environmental selection and enrichment of microbes in dammed environments. Similarly, previous studies have also demonstrated strong effect of depth on microbial structure (Fierer et al., 2003; Allison et al., 2007; Eilers et al., 2012; Leewis et al., 2022). The distinctness of microbial distribution at depths was highlighted through the associations between major microbial taxa and environmental parameters (Figure 3). This study confirmed our previous observation and speculations that soil moisture, redox, Fe, carbon and nitrogen content play key roles in shaping the microbiome structure and distribution across depth in legacy sediments (e.g., Figures 3C,D; Sienkiewicz et al., 2020). All these matrices are limiting factors influencing and shifting the microbial assembly and distribution (e.g., Fierer and Jackson, 2006; Fierer, 2017; Weitzman and Kaye, 2017; Jansson and Hofmockel, 2020).

The microbiomes (both bacteria/archaea and fungi) differed between watersheds (Supplementary Figure S3), reflecting the impact from local land use, geology and sediment lithology, and concurrent stream conditions (Sienkiewicz et al., 2020). Chiques Creek is an agriculture stream running through Lancaster County in Pennsylvania while the Cooch dam site is a more urbanized site near Newark in Delaware. Previous studies for the Roller and Cooch riparian sediments indicated significant differences in biogeochemistry (Inamdar et al., 2022; Peck et al., 2022, 2023). The sediment samples from Chiques Creek contained higher %C, %N, nitrate-N, and metals (Cu, Zn, Ca etc.) indicating the influence of agriculture inputs and sediment lithology (Peck et al., 2022, 2023). In contrast, Cooch sediments contained higher Na and Fe concentrations, with the elevated Na concentrations likely due to road salt applications (Inamdar et al., 2022; Peck et al., 2023). Thus, microbiome fingerprints in riparian legacy sediments upstream of the dams reflect human activities, watershed conditions, as well as land uses (Bissett et al., 2014; Ding et al., 2022).

### Impact of dam removal on microbial assembly and function

**Figure 5:**
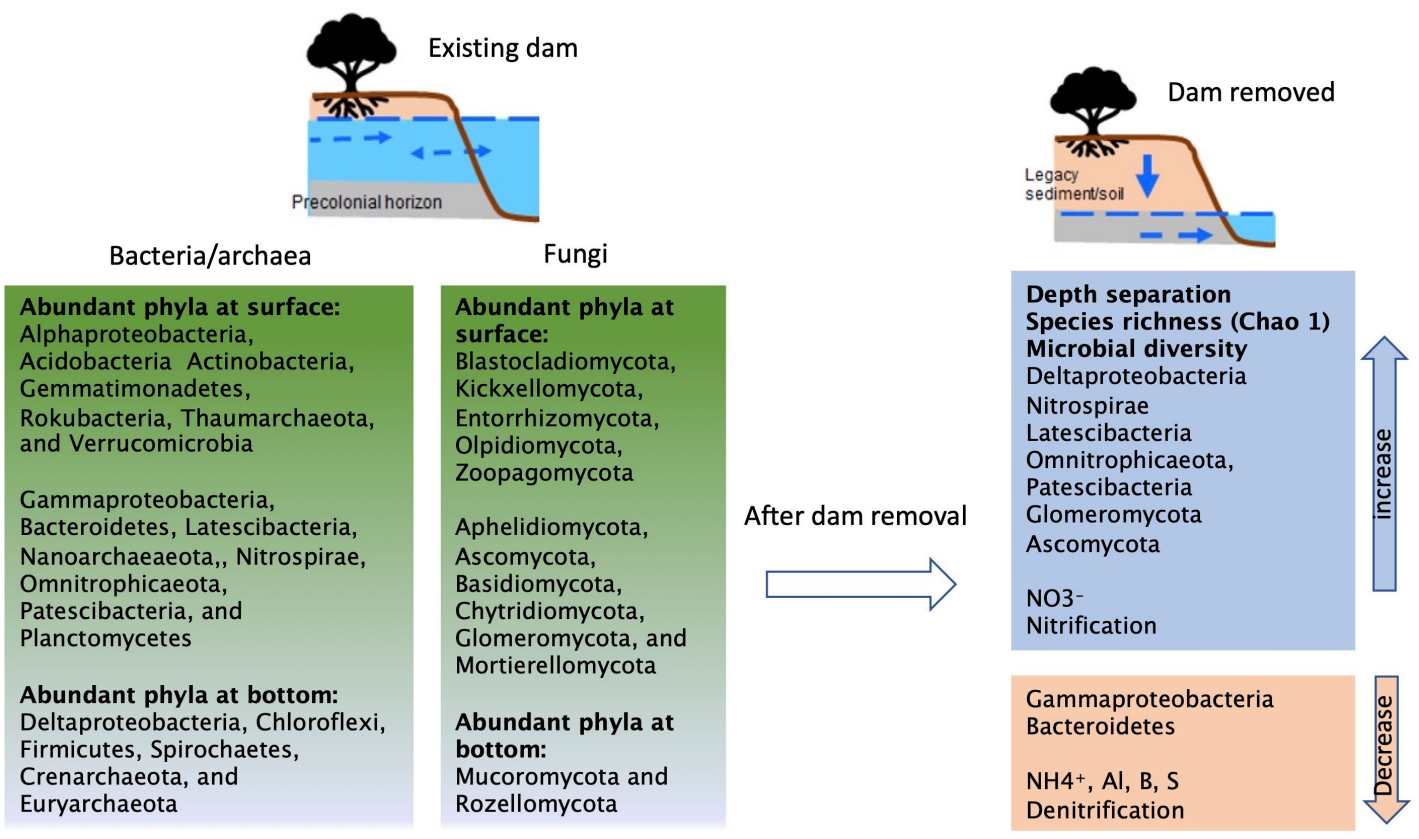
A schematic diagram summarizing the potential impacts of dam removal on microbial assembly and function.

A schematic diagram based on our observations of changes before and after dam removal was summarized in Figure 5. In the presence of the milldam, upstream riparian sediments are saturated and persistently hypoxic or anoxic, especially below 1 m depth (Inamdar et al., 2022). Sherman et al. (2022) also found that groundwaters in the near-stream riparian zones at Roller and Cooch were poorly mixed. In contrast, following dam removal (as in case of Krady), the sediments drain rapidly and become oxic in a few hours or days (Lewis et al., 2021). The hydrological connectivity and mixing impacts soil moisture and redox gradients, and therefore drive and shift microbial community composition and related biogeochemical processes (Argiroff et al., 2017). For instance, high moisture content is likely associated with greater microbial biomass (Serna-Chavez et al., 2013; Fierer, 2017). Increases in soil moisture level affect nutrient connection and cycling and therefore impact microbial interactions and population structure (Barnard et al., 2013; Jansson and Hofmockel, 2020). Along with groundwater fluctuation, redox changes also play import roles in manipulating microbial assembly in many different ways such as navigation of energy taxis (Alexandre et al., 2004) and shifts between active and inactive biomass (Khurana et al., 2022). Increased abundances of Deltaproteobacteria (Figures 3A, 4A) such as iron reducing bacteria Geobacter and Fe^II^ concentrations at the depths also indicate reducing conditions (Childers et al., 2002; Duval and Hill, 2007; Lutgen et al., 2020). Following hydrological disruption and redox kinetics, further changes in carbon and nitrogen processing will gradually and continuously alter microbial composition and their functional properties (Fierer and Jackson, 2006; Kaushal et al., 2008; Argiroff et al., 2017; Weitzman and Kaye, 2017).

In this study, we were not able to collect samples before the breach of Krady dam, but we used the Roller dam, located 10 km upstream, as comparison to represent conditions in the presence of the dam. Our results clearly revealed important differences between sediments from the Krady vs. Roller site (Figures 4, 5). After dam removal, higher species richness and diversity, Nitrospirae, Deltaproteobacteria, Glomeromycota, and Ascomycota were found at the Krady site. The higher abundance of Acidobacteria and Verrucomicrobia in Krady sediments, post dam removal, could be associated with the more dynamic riparian groundwater regime which allowed for greater variation of soil moisture with rewetting of the riparian soils from rainfall and flood events. This would agree with observations from Barnard et al. (2013), who showed that these two bacterial phyla increased with re-wetting events. Gammaproteobacteria and Bacteroidetes were more abundant at the Roller site (existing dam) with persistently saturated soil conditions, suggesting they are possibly sensitive to drying conditions, which corroborate the previous results and observations (Zeglin et al., 2011; Barnard et al., 2013). In comparison, Actinobacteria are more tolerant to desiccation and favor in drying conditions or low humidity (Barnard et al., 2013; Barka et al., 2016). For this study, Actinobacteria was abundant in surface sediments but did not differ between the Krady and the Roller sites, suggesting that the microbial distribution could also be affected by other environmental factors. Lastly, since the samples were not collected at the same dam site (Krady) before and after dam removal, we could not completely exclude the potential for spatial heterogeneity between the two sites. But based on our previous survey, the differences between the adjacent sites on the same stream and within the same watershed should be minor (Sienkiewicz et al., 2020).

Nitrification/denitrification and their associated genes are the most sensitive pathways in response to water level fluctuations (Zhang et al., 2021). Our results support the hypothesis that dam removal enhances nitrification process (Figure 4C). Higher nitrification separated the samples from Krady site, where they were taken 16 months after the dam breached (Lewis et al., 2021). We also hypothesized that the dam removal inhibited denitrification due to water drop with loss of saturation and anoxic conditions (Barnard et al., 2013; Jansson and Hofmockel, 2020; Lewis et al., 2021). However, neither amended nor unamended DEA showed strong correlations with the microbial distributions. Meanwhile, higher concentrations of NO_3_-N and δ^15^N supported reduced denitrification in sediment samples after the dam removal (Lewis et al., 2021). Lewis et al. further confirmed that denitrification in riparian sediments did decrease after dam removal, but the N concentrations in groundwaters and stream waters did not increase (Lewis et al., 2021). Low DEA measurements contradicted the quantification of denitrifying genes in this study, and could be attributed to dormant denitrifying microorganisms in these sediments (Jones and Lennon, 2010). The qPCR approach we used did not differentiate between active vs. dormant denitrifiers. In addition, the fact that the anoxic sediments behind standing dam at Roller site contained higher concentrations of ammonium-N and lower nitrate-N (Figure 4; Inamdar et al., 2022) led us to infer that other nitrogen processes (such as dissimilatory nitrate reduction to ammonia, DNRA) could be influencing denitrification and associated microbial communities in the deeper sediments (Pandey et al., 2020; Wang et al., 2020; Inamdar et al., 2022; Peck et al., 2022). All these potential pathways and processes warrant further investigations. In fact, microbial communities and process rates for denitrification and DNRA associated with milldam sediments are currently being investigated in our ongoing projects and incubation experiments.

## Conclusion

Taken together, our findings reveal important differences in the composition and distribution of bacteria/archaea and fungi in riparian legacy sediments above dams. Both prokaryotic communities and fungi show clear spatial patterns across watersheds and sediment depths. In addition to vertical gradients, microbial community assembly and distribution are also driven by location and co-current conditions, suggesting the microbiomes in the accumulated sediments record past human activities and contemporary land uses. Dam removal and associated changes in hydrologic and biogeochemical regimes reinforce depth distribution of soil microbiomes and influence microbial diversity, composition, and function. Our data support that dam removal enhanced nitrification. The discrepancy between the measured denitrification enzyme assay (DEA) and the quantification of *nos*Z genes (via qPCR) indicates the occurrence of dormant denitrifying microbes or other nitrogen-competing processes such as DNRA. Further research into how microbial communities and functions change after dam removal will improve our understanding of microbial ecology in fragmented river systems. Results will also provide valuable information and guidance to stakeholders and restoration projects.

## Data availability statement

The datasets presented in this study can be found in the article or Supplementary material. Sequencing data obtained in this study are available through the GenBank database under Bioproject number PRJNA925921: https://www.ncbi.nlm.nih.gov/sra?LinkName=bioproject_sra_all&from_uid=925921.

## Author contributions

JK and SI: conceptualization. EP and SI: experimental operation. JK and LZ: data analysis. JK: writing—original draft preparation. EP, LZ, MP, and SI: writing—reviewing and editing. JK, MP, and SI: funding acquisition. All authors contributed to the article and approved the submitted version.

## Funding

This study was supported by NSF (NSF-HS-1929747, NSF-EAR-2213856), USDA 2020-67019-31164, and Endowment Fund of Stroud Water Research Center.

## Conflict of interest

The authors declare that the research was conducted in the absence of any commercial or financial relationships that could be construed as a potential conflict of interest.

## Publisher’s note

All claims expressed in this article are solely those of the authors and do not necessarily represent those of their affiliated organizations, or those of the publisher, the editors and the reviewers. Any product that may be evaluated in this article, or claim that may be made by its manufacturer, is not guaranteed or endorsed by the publisher.

## Footnotes

^1^https://support.illumina.com/

^2^https://github.com/qiime2/q2-featureclassifier

^3^https://github.com/qiime2/q2-diversity
