# Supplementary material for: Mill dams impact microbiome structure and depth distribution in riparian sediments
Source: Front Microbiol. 2023 Jun 29;14:1161043. doi: 10.3389/fmicb.2023.1161043 (PMC10339028; doi:10.3389/fmicb.2023.1161043)
Supplement: Supplementary file 2 [file Data_Sheet_1.docx]

Supplementary materials

**Mill dams impact microbiome structure and depth distribution in riparian sediments**

Jinjun Kan^1*^, Erin K. Peck^2,#^, Laura Zglesweski^1^, Marc Peipoch^1^, and Shreeram Inamdar^2^

^1^ Stroud Water Research Center, Avondale, PA 19311, United States

^2^University of Delaware, Plant & Soil Sciences, Newark, DE 19716, United States

*Correspondence: Jinjun Kan, jkan@stroudcenter.org

# Current address: University of Massachusetts Amherst, Northeast Climate Adaptation Science Center, Amherst, MA 01002, United States

**Fig. S1** Rarefaction curves showing the sampling depth with the coverage of microbial diversity in our sequencing samples. With both cutoffs for bacteria (left) and fungi (right), we are able to cover >99.7% of the total diversity. Rarefaction curves were constructed using the 'alpha-rarefaction' visualizer in the qiime2 q2-diversity plugin (<https://github.com/qiime2/q2-diversity>) using the Goods coverage method. The ASV table was then rarefied using the qiime2 q2-feature-table plugin with the 'rarefy' method (<https://github.com/qiime2/q2-feature-table>).

**Fig. S2** Standard curves to quantify the copy numbers of AOA *amo*A genes (top) and *nos*Z genes (bottom).


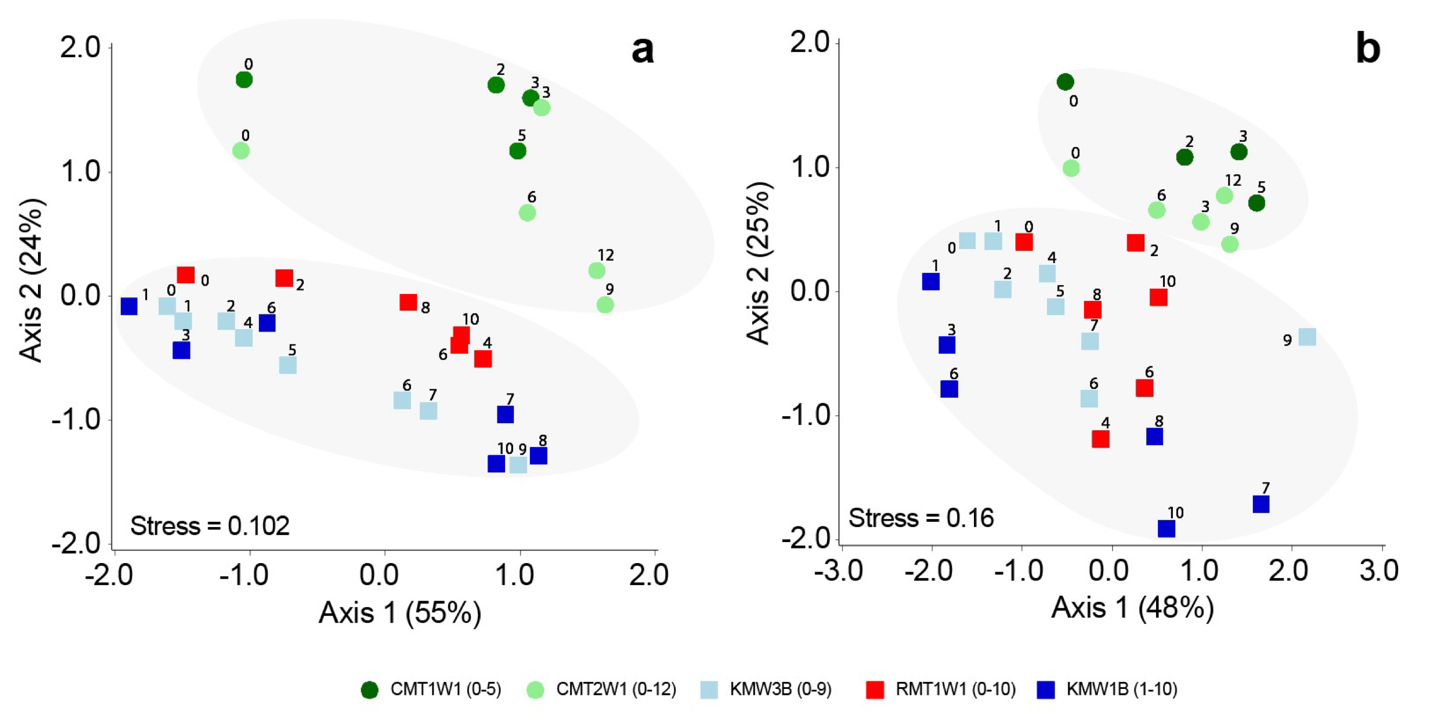


**Fig. S3** NMDS plots to show bacteria/archaea (a) and fungi (b) community structures across depths from all dam sites. Cooch site is on Christina River; Krady and Roller dam sites are on Chiques Creek.
